# Comparison of Effects of General Versus Spinal Anesthesia on Spermiogram Parameters and Pregnancy Rates After Microscopic Subinguinal Varicocelectomy Surgery: Retrospective Cohort Analysis

**DOI:** 10.3390/medicina62010133

**Published:** 2026-01-08

**Authors:** Levent Özdemir, Aslınur Sagün, Mert Başaranoğlu, Elif Tuna Sevim, Mustafa Azizoğlu, Erdem Akbay

**Affiliations:** 1 Department of Anesthesiology and Intensive Care, Mersin University Faculty of Medicine, Mersin 33110, Türkiye; aslinur_aslan@hotmail.com (A.S.); eliftunasev@mersin.edu.tr (E.T.S.); dryalamaoglu@hotmail.com (M.A.); 2 Department of Urology, Mersin University Faculty of Medicine, Mersin 33110, Türkiye; mertbasaranoglu@gmail.com (M.B.); erakbay@mersin.edu.tr (E.A.)

**Keywords:** infertility, spermiogram, varicocelectomy, spinal anesthesia, general anesthesia, pregnancy rate

## Abstract

*Background and Objectives:* The association between different anesthesia modalities and spermiogram parameters and reproductive outcomes in patients undergoing microscopic subinguinal varicocelectomy (MSV) remains unclear. In this retrospective cohort study, we aimed to compare spermiogram parameters and pregnancy rates between patients receiving general anesthesia (GA) versus spinal anesthesia (SA) for MSV with 2-year follow-up data. *Materials and Methods:* Male patients aged between 18–50 years, with ASA physical scores between I–III, who underwent unilateral or bilateral primary MSV, were included in the study. To minimize selection bias and balance the baseline characteristics between the GA group and SA group, we employed a propensity score matching approach, matching all 38 SA patients with 380 GA patients selected from a larger pool. Patients with complete 24-month follow-up data were included in the final analysis. The primary outcome of our study was determined as evaluating sperm count changes. Secondary outcomes included other sperm parameters (motility, morphology and semen volume), natural pregnancy rates, perioperative complications and recovery parameters. *Results:* The final analysis included 418 patients who met all inclusion criteria and completed the follow-up period. The study population comprised 380 patients in the GA group and 38 in the SA group. No significant difference was found between the groups in terms of sperm count. Greater improvement in sperm motility was observed in the SA group starting from the third month onwards (*p* = 0.027). Natural pregnancy was achieved in 16/38 (42.1%) of SA patients versus 125/380 (32.9%) of GA patients (*p* = 0.031). In addition, better results were obtained in terms of recovery parameters in the SA group. Other results were comparable between the groups. *Conclusions:* Spinal anesthesia for MSV was associated with greater improvement in sperm motility and higher natural pregnancy rates compared to general anesthesia, despite comparable sperm count improvements. These associations warrant further investigation in prospective randomized trials.

## 1. Introduction

Varicocele is an abnormal dilation of the testicular venous pampiniform plexus and is one of the most common correctable causes of male infertility [1]. It occurs in approximately 15% of men and its prevalence in infertile men may be as high as 40% [2]. Varicocele may impair sperm quality through impaired perfusion, testicular hyperthermia, increased oxidative stress, and hormonal imbalances [3,4].

Microscopic subinguinal varicocelectomy (MSV) is one of the frequently preferred methods in the surgical treatment of this pathology [5]. The type of surgical procedure is the most decisive factor in selecting the appropriate anesthesia technique, but it also depends on various factors such as patient safety, perioperative pain control, time to return to normal activity, and cost-effectiveness [6]. Although many studies have documented the clinical efficacy of MSV and its positive improvements to sperm tests [7,8,9,10], the effects of different anesthesia modalities on spermiogram parameters and reproductive outcomes in patients undergoing MSV remain unclear.

MSV can be successfully performed under general anesthesia (GA), spinal anesthesia (SA) or local anesthesia. Systemic effects of anesthetic agents have been associated with mechanisms similar to surgical stress response, vasomotor changes, and ischemia–reperfusion injury [11]. The effects of inhalational agents leading to oxidative stress have been reported in previous studies [12]. General anesthetics may also reduce testosterone levels due to their ability to temporarily suppress the hypothalamic-pituitary-gonadal axis [13]. In addition, general anesthesia may cause testicular hypoperfusion by systemic vasodilation. Local anesthetics administered in central neuraxial blocks have minimal systemic effects and may suppress the surgical stress response and may also improve testicular blood flow by reducing sympathetic tone.

When the current literature is reviewed, there are very few studies evaluating the direct effects of the type of anesthesia on sperm parameters and natural pregnancy rates. The existing studies generally focus on the feasibility of surgery under local anesthesia and recovery parameters [6,14]. Furthermore, long-term follow-up data required to obtain important results are lacking.

In this retrospective cohort study, we aimed to compare the effects of general versus spinal anesthesia on spermiogram parameters and pregnancy rates after MSV with 2-year follow-up data.

## 2. Materials and Methods

### 2.1. Ethical Approval

This retrospective cohort study was conducted at Mersin University Hospital (in Mersin, Türkiye) between January 2018 and March 2023. The study received approval from the Mersin University Clinical Research Ethics Committee with decision number 2025/239 dated 5 March 2025, and was conducted in accordance with the principles of the Declaration of Helsinki. Clinical trial registration was not applicable due to the retrospective study design.

### 2.2. Study Design and Patient Selection

This study included male patients aged 18 to 50 who underwent unilateral or bilateral primary microsurgical subinguinal varicocelectomy (MSV) and were operated on solely for male infertility. To minimize confounding from female factors, we included only couples where female partners (all female partners were aged <40 years old and mean age was 31.2 ± 4.8) had documented regular menstrual cycles, patent fallopian tubes (confirmed by hysterosalpingography or laparoscopy), and normal ovarian reserve (anti-Müllerian hormone > 1.0 ng/mL and antral follicle count > 5). Female partners with diagnosed endometriosis, polycystic ovary syndrome, used ART or prior pelvic surgery were excluded. Patients who had surgery completed under spinal or general anesthesia and who were not converted from spinal to general anesthesia for any reason were included in the study. In the routine practice of our hospital, varicocele grading is done according to the Dubin-Amelar classification system and confirmed by two independent urologists. Scrotal Doppler ultrasonography (Philips EPIQ 7G, Eindhoven, The Netherlands) was performed using a high-frequency linear transducer (5–12 MHz). The diagnostic criteria included vein diameter ≥3.0 mm, retrograde flow lasting >1 s, and flow velocity > 2 cm/s during valsalva maneuver.

Semen analysis was performed after 2–5 days of sexual abstinence, as per WHO guidelines. Exclusion criteria included patients converted from spinal anesthesia to general anesthesia, recurrent varicocele, combined surgical procedures, follow-up duration < 2 years, ASA physical status IV or higher, incomplete medical records, chronic medication use affecting spermatogenesis (e.g., finasteride, exogenous androgens, SSRIs), and the presence of conditions affecting spermatogenesis (e.g., cryptorchidism, hypogonadism, testicular trauma, chemotherapy).

### 2.3. Propensity Score Matching Methodology

**Figure 1:**
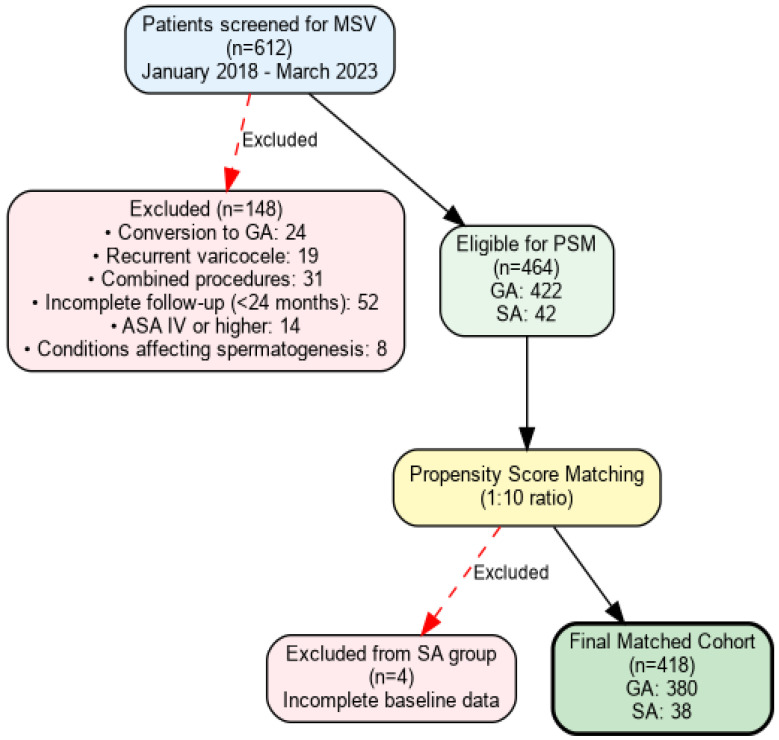
Flowchart of patient selection. The diagram illustrates the complete patient selection process for the study, showing the initial screening of 612 patients who underwent microscopic subinguinal varicocelectomy (MSV) between January 2018 and March 2023. After applying inclusion and exclusion criteria, the final matched cohort consisted of 418 patients (380 general anesthesia, 38 spinal anesthesia) using a 1:10 matching ratio.

Initial screening identified 612 patients who underwent MSV during the study period. After applying inclusion and exclusion criteria, 464 patients remained eligible: 422 in the GA group and 42 in the SA group. The final matching process resulted in 38 SA patients matched with 380 GA patients, maintaining the complete SA cohort while selecting the most comparable GA patients (Figure 1).

This methodology helps mitigate the potential impact of selection bias while maximizing the use of available data. Balance diagnostics were performed to ensure the success of the matching procedure, including visual inspection of propensity score distributions and standardized difference plots for all covariates. Additionally, sensitivity analyses were conducted to assess the robustness of our findings to unmeasured confounding using Rosenbaum bounds method. The analysis revealed that an unmeasured confounder would need to increase the odds of receiving spinal anesthesia by a factor of 1.8 (Gamma = 1.8, *p* = 0.042) to invalidate the significance of the sperm motility outcome, and by a factor of 1.6 (Gamma = 1.6, *p* = 0.038) for pregnancy rate, indicating reasonable resistance to hidden bias. The smaller SA cohort reflected patient and surgeon preference for GA at our institution.

To minimize selection bias and balance the baseline characteristics between the general anesthesia (GA) and spinal anesthesia (SA) groups, we employed a 1:10 propensity score matching approach. This ratio was chosen to retain all SA patients while selecting comparable GA patients from the larger pool, as matching ratios up to 1:10 can improve precision when the control group is substantially larger. The propensity score was calculated using a logistic regression model that incorporated key covariates known to influence treatment selection and outcomes, including age (±2 years), body mass index (±2 kg/m^2^), ASA physical status, varicocele grade, bilateral versus unilateral involvement, duration of infertility, baseline sperm parameters, and preoperative vein diameter measurements.

For each patient in the SA group (*n* = 38), we matched ten patients from the GA group with the closest propensity score using a caliper width of 0.2 standard deviations of the logit of the propensity score. This approach, as described by Desai et al. allows for optimal balance between groups while preserving statistical power [15]. The quality of matching was assessed through standardized mean differences (SMD), with values < 0.1 considered indicative of good balance. All standardized mean differences (SMD) for matched covariates were <0.1, confirming excellent balance: age (SMD = 0.062), BMI (SMD = 0.089), ASA status (SMD = 0.074), varicocele grade (SMD = 0.058), baseline sperm count (SMD = 0.033), motility (SMD = 0.007), morphology (SMD = 0.041), and infertility duration (SMD = 0.052).

### 2.4. Anesthetic Management and Surgical Technique

Data on patients who underwent two different anesthesia techniques were collected from patient files and hospital automation system. Patients with missing data were excluded from the study.

GA was induced with propofol (2–3 mg/kg) and fentanyl (1–2 μg/kg), followed by rocuronium (0.6 mg/kg) for endotracheal intubation. Anesthesia was maintained with sevoflurane (1.5–2.5% end-tidal concentration) in O_2_-N_2_O mixture (FiO_2_ 0.4–0.5). Additional fentanyl boluses (0.5–1 μg/kg) were administered as needed for analgesia. Neuromuscular blockade was reversed with neostigmine (0.04 mg/kg) and atropine (0.02 mg/kg) at the end of surgery.

SA was performed at the L3–L4 or L4–L5 interspace using a 25–27 gauge pencil point needle with the patient in sitting position. Heavy bupivacaine 0.5% (12.5–15 mg) was administered intrathecally without additives. Sensory block level was assessed to T6–T10 before surgical incision. No intraoperative sedation was administered to SA patients to avoid confounding effects on reproductive outcomes.

All MSV procedures were performed by two experienced urologic surgeons using standardized technique. A 2–3 cm subinguinal incision was made 1–2 cm below the external inguinal ring. The spermatic cord was delivered and examined under 10–25× magnification (Carl Zeiss OPMI Pentero 900, Jena, Germany). Internal spermatic veins were identified, isolated, and ligated with 4-0 silk sutures while preserving the testicular artery, lymphatics, and vas deferens. The cremasteric vein was also ligated. Testicular artery pulsations were confirmed with Doppler ultrasonography before closure.

Intraoperative paracetamol 10 mg/kg was routinely administered 30 min before the end of surgery in both groups. Postoperatively, multimodal analgesia included scheduled paracetamol (1 g every 6–8 h) and rescue intravenous morphine (0.01–0.02 mg/kg) if VAS score > 3. Postoperative opioid consumption was recorded during the first 24 h. Patients who developed intraoperative bradycardia, hypotension or bleeding were identified according to predefined criteria (Section 2.5). Postanesthesia care unit and urology ward records were used for postoperative complications.

### 2.5. Data Collection and Outcome Measures

The primary outcome of our study is to evaluate the impact of various anesthesia methods on sperm count. Secondary outcomes include their effects on other sperm parameters (such as motility, morphology and semen volume), pregnancy rates, recovery outcomes and postoperative complications.

In our hospital, semen analysis was performed using Computer-Aided Sperm Analysis (CASA) system (SCA^®^ CASA System v6.2, Microptic S.L., Barcelona, Spain), calibrated monthly according to manufacturer specifications. Laboratory conditions were strictly maintained at 22 ± 2 °C temperature and 50 ± 5% humidity, monitored using calibrated environmental sensors (Testo 608-H1, Germany). A comprehensive quality control program was implemented throughout the study period. This included internal quality control using commercial control samples and monthly external quality assessment through participation in the NEQAS scheme.

All analyses were performed in an ISO 15189 (defines the quality and competency requirements for medical laboratories as set by the International Organization for Standardization) accredited andrology laboratory, with sperm morphology assessment following strict Kruger criteria using Diff-Quik staining protocol. Samples were processed within 30 min of collection in a Class II biological safety cabinet, and the laboratory maintained its quality standards through bi-annual proficiency testing via the CAP External Quality Assurance Scheme.

Sperm parameters were evaluated using standardized protocols at baseline and during scheduled follow-up visits at 1, 3, 6, 12, and 24 months postoperatively. Analysis included sperm count (million/mL, assessed using WHO-approved hemocytometer), motility (% progressive motility, computer-assisted sperm analysis), morphology (% normal forms, strict Kruger criteria), and semen volume (mL, calibrated containers). All semen analyses were performed in accordance with WHO laboratory guidelines (5th edition, 2010) by experienced andrologists blinded to the anesthetic technique.

Natural pregnancy was defined as spontaneous conception confirmed by positive serum β-hCG (>25 mIU/mL) or transvaginal ultrasound demonstrating intrauterine gestational sac with fetal cardiac activity at 6–8 weeks gestation, without use of assisted reproductive technologies. Pregnancy outcomes were systematically collected from hospital obstetric records over a 24-month period following surgery.

Complications were recorded and categorized using standardized definitions. Intraoperative events included bradycardia (heart rate < 50 bpm), hypotension (mean arterial pressure < 50 mmHg), and bleeding (>50 mL). Postoperative complications comprised post-dural puncture headache (as per International Headache Society criteria), nausea/vomiting, urinary retention (requiring catheterization), and scrotal hematoma. Recovery parameters including time to ambulation, hospital stay duration, and pain scores at 6 and 24 h were systematically documented.

### 2.6. Statistical Analysis

Sample size calculation was performed a priori using G*Power 3.1.9.7 (Heinrich-Heine-Universitat Dusseldorf, Germany), assuming an effect size of 0.5 for the primary outcome (sperm count improvement), alpha of 0.05, and power of 0.8. This calculation indicated a minimum requirement of 64 patients per group to detect clinically significant differences. The SA group (*n* = 38) falls below this threshold due to the retrospective design and limited number of patients who received SA during the study period. Consequently, this study may be underpowered for detecting small-to-moderate effect sizes in the SA group. We therefore emphasize effect sizes and confidence intervals rather than solely relying on *p*-values, and interpret findings with appropriate caution regarding Type II error risk.

Statistical analyses were performed using Python version 3.9.7 (Python Software Foundation) with scipy v1.7.1 and statsmodels v0.13.0. For each sperm parameter, the percentage improvement was calculated using the standardized formula: [(postoperative value − preoperative value)/preoperative value] × 100. Effect size calculations utilized Cohen’s d, with values of 0.2, 0.5, and 0.8 representing small, medium, and large effects, respectively, based on established conventions in reproductive medicine research. Risk ratios with 95% confidence intervals were calculated for categorical outcomes. The homogeneity of variances was confirmed using Levene’s test, and multiple comparison correction was performed using the Bonferroni method to maintain family-wise error rate at 0.05. Temporal changes in sperm parameters were analyzed using mixed-effects models to account for repeated measurements, with post hoc comparisons at each time point adjusted for multiple testing. Achievement of WHO reference values was assessed for each parameter and compared between groups. Statistical significance was set at *p* < 0.05.

## 3. Results

### 3.1. Patient’s Demographics and Baseline Characteristics

The final analysis included 418 patients who met all inclusion criteria and completed the follow-up period. The study population comprised 380 patients in the general anesthesia (GA) group and 38 in the spinal anesthesia (SA) group. Baseline demographic and clinical characteristics demonstrated comparable distribution between groups (Table 1). All preoperative sperm parameters were statistically similar between the cohorts.

### 3.2. Perioperative Outcomes and Complications

Detailed analysis of perioperative events revealed distinct patterns between groups (Table 2). Mean surgery duration was comparable between groups (58.3 ± 12.1 min for GA group vs. 60.2 ± 10.4 min for SA group, *p* = 0.161). In the GA cohort, intraoperative complications included bradycardia in 19 patients (5.0%, 95% CI: 3.0–7.7%), hypotension in 24 patients (6.3%, 95% CI: 4.1–9.2%), and bleeding exceeding 50 mL in 13 patients (3.4%, 95% CI: 1.8–5.8%). The SA group demonstrated a comparable safety profile, with bradycardia in 3 patients (7.9%, 95%CI: 1.7–21.4%) and hypotension in 1 patient (2.6%, 95%CI: 0.1–13.8%), with no bleeding complications reported. Most patients in both groups remained complication-free (GA: 85.3%, SA: 89.5%, RR: 1.05, 95% CI: 0.94–1.17).

### 3.3. Recovery Parameters

Recovery parameters significantly favored the SA group, with shorter time to ambulation (4.1 ± 1.2 vs. 6.2 ± 1.8 h, *p* < 0.001) and reduced length of hospital stay (24.7 ± 5.1 vs. 28.4 ± 6.3 h, *p* = 0.002). Total postoperative morphine consumption in the first 24 h was significantly lower in the SA group (2.8 ± 3.2 mg) compared to the GA group (8.4 ± 5.7 mg, *p* < 0.001). Pain scores were consistently lower in the SA group at both 6 h (2.1 ± 0.9 vs. 3.8 ± 1.4, *p* < 0.001) and 24 h (1.7 ± 0.6 vs. 2.3 ± 0.8, *p* = 0.004) postoperatively (Table 2). However, the SA group experienced a higher incidence of urinary retention (15.8% vs. 1.8%, RR: 8.77, 95%CI: 3.12–24.65, *p* < 0.001) and exclusive occurrence of post-dural puncture headache (7.9%, *p* < 0.001).

### 3.4. Comparison of Pre-Versus Postoperative Spermiogram Parameters and Pregnancy Rates

**Figure 2:**
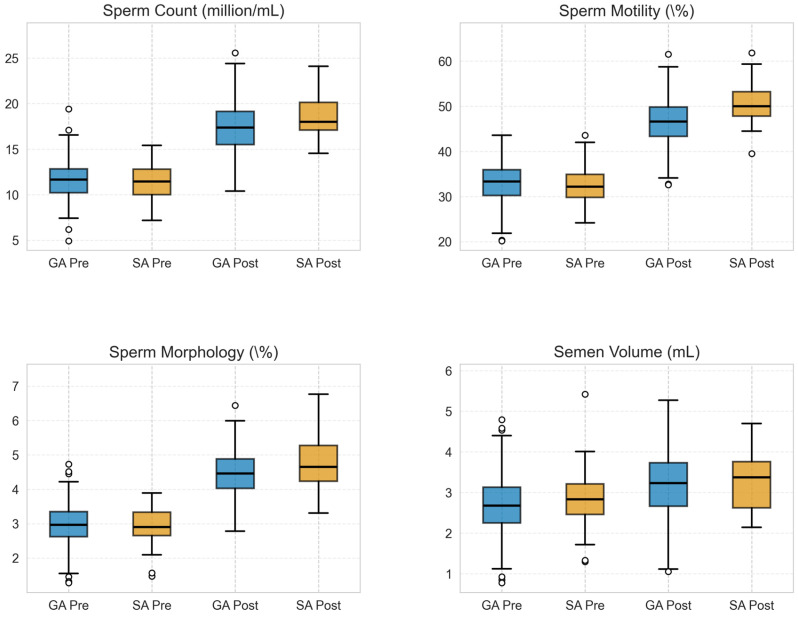
Distribution of sperm parameters before and after surgery. Box plots showing the distribution of sperm parameters in GA and SA groups, both pre- and post-operatively.

Detailed box plot analysis (Figure 2) confirmed the comparability of preoperative value distributions between groups, validating baseline homogeneity. While outliers were identified, particularly in the general anesthesia group, sensitivity analyses confirmed that these did not significantly influence the overall results.

**Figure 3:**
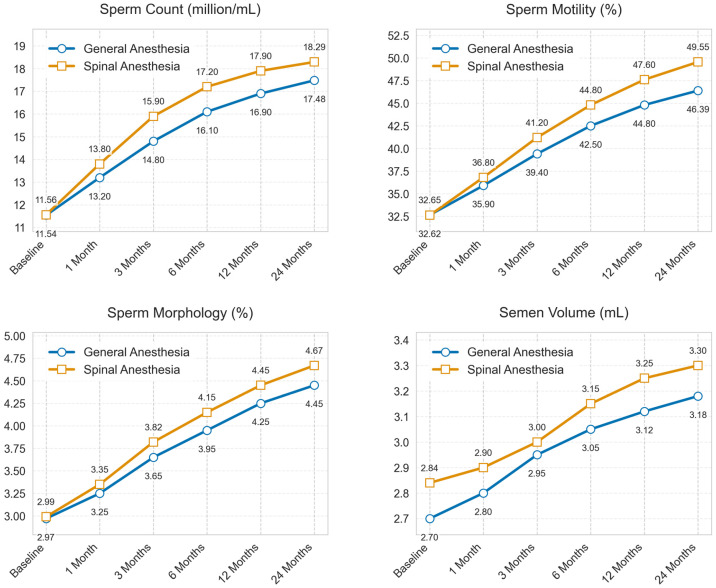
Temporal changes in sperm parameters over 24-month follow-up. Line graphs demonstrating the temporal evolution of sperm parameters over a 24-month period. General anesthesia group is marked with a blue circle and the spinal anesthesia group is marked with a red square.

Longitudinal analysis over the 24-month follow-up period revealed distinct patterns of improvement (Figure 3). Both groups showed progressive enhancement in sperm parameters, with the most substantial gains observed within the first 6 months. The SA group demonstrated consistently superior improvement trajectories in sperm motility, where the difference became apparent from month 3 onwards and continued to widen throughout the follow-up period (*p* = 0.027) (Table 3). The groups were comparable in terms of sperm count, morphology and semen volume, and no statistically significant difference was found between the groups (*p* > 0.05).

## 4. Discussion

In this retrospective cohort study, we examined the association between anesthesia modality (general versus spinal) and spermiogram parameters and natural pregnancy rates following microscopic subinguinal varicocelectomy (MSV), with 24-month follow-up data. No significant difference was found between the groups in terms of sperm count, which was our primary outcome. Patients receiving spinal anesthesia demonstrated numerically greater improvement in all sperm parameters, with the only statistically significant difference observed in sperm motility from month 3 onwards. Natural pregnancy rates at 24-month follow-up were significantly higher in the spinal anesthesia group (42.1% vs. 32.9%, *p* = 0.031). Additionally, the spinal anesthesia group showed more favorable recovery parameters. Other results were comparable between the groups.

When the literature is reviewed, there is no study comparing the effects of spinal and general anesthesia on semen analysis and natural pregnancy rates in MSV. The studies that have been conducted have mostly focused on the success of the surgical technique, postoperative pain and recovery scores depending on the anesthesia types [6,16]. Some studies have focused on the feasibility of MSV under local anesthesia and the postoperative discharge process [14].

Some trials conclude that the subinguinal approach leads to the best outcomes [17], while others favor the inguinal approach [18]. Although different surgical techniques have been described, MSV has become the most preferred surgical option due to its advantages such as meticulous ligation of all vessels, preservation of arterial branches, lower postoperative recurrence rate, fewer complications, higher postoperative semen quality and higher postoperative fertility rate [19]. The advantage of the subinguinal approach is that it does not require dissection of the external abdominal oblique muscle and includes shallow spermatic cord and simple anatomical layers. This can result in minor surgical trauma and shorter recovery time and is one of the important factors in its preference [20]. For this reason, whether it can be performed under local anesthesia has been the subject of some studies. Although there are some advantages of performing MSV under local anesthesia, it has also been reported that intraoperative VAS scores are higher [16], thus sometimes requiring deep sedation and causing higher anxiety scores in patients. When evaluated from this perspective, MSV may be appropriate to perform under spinal anesthesia when it is desired to avoid the systemic effects of general anesthetic agents.

Several plausible mechanisms may explain the observed association between anesthesia modality and sperm motility outcomes. General anesthesia is associated with a systemic stress response that may provoke oxidative stress and transient hormonal imbalances [11,12,13]. Volatile anesthetics, particularly sevoflurane used in our protocol, have been shown to increase reactive oxygen species production and lipid peroxidation in various tissues [12]. While spermatogenesis requires approximately 74 days in humans, sperm maturation in the epididymis continues for 2–3 weeks, during which sperm acquire progressive motility. The observed motility differences emerging at 3 months post-surgery align with the timeline of sperm that were in the epididymal maturation phase at the time of surgery, potentially exposing them to perioperative systemic effects.

An additional consideration in our general anesthesia protocol is the use of nitrous oxide (N_2_O), which has been associated with potential reproductive toxicity in several studies [21,22]. N_2_O interferes with vitamin B12 and folate metabolism by irreversibly oxidizing the cobalt center of vitamin B12, which may impair DNA synthesis and methionine synthase activity. Chronic occupational exposure to N_2_O has been linked to reduced fertility and increased miscarriage risk [21]. The authors found that the effect was evident only in the 19 women with five or more hours of exposure per week [21]. While our patients received shorter intraoperative exposure (typically 60 min), the potential effect on sperm parameters recovering during the postoperative period cannot be entirely excluded. This represents an additional confounding factor that may have contributed to the observed differences between groups and should be acknowledged as a limitation of our study. Future studies comparing anesthesia modalities in fertility procedures should consider N_2_O-free general anesthesia protocols to isolate the effects of other anesthetic agents.

Another factor that could affect the results is that general anesthetics can temporarily suppress the hypothalamic-pituitary-gonadal axis [13], which may alter testosterone and gonadotropin levels for days to weeks after surgery. Although we did not measure hormonal profiles longitudinally, this temporary suppression could theoretically impact the final stages of sperm maturation. Conversely, spinal anesthesia provides testicular sympathetic blockade, which may enhance testicular perfusion during and immediately after surgery, potentially creating a more favorable microenvironment for recovering spermatogenesis. The reduced systemic opioid requirements in the SA group (as evidenced by lower pain scores with similar analgesic protocols) may also contribute, as opioids can suppress gonadotropin secretion [13]. However, it is important to emphasize that these remain hypothetical mechanisms. The retrospective design and absence of mechanistic biomarkers (oxidative stress markers, hormonal profiles, sperm DNA fragmentation) limit our ability to confirm causality. The observed associations could also reflect unmeasured confounding despite propensity score matching.

Although direct comparisons in the context of varicocelectomy are sparse, assisted reproductive technology (ART) studies offer some insights [23,24]. Improved outcomes in these settings are thought to arise from diminished exposure to anesthetic-induced oxidative stress and a lowered neuroendocrine stress response during the procedure. Studies comparing anesthesia modalities in ART have provided evidence suggesting that spinal anesthesia may be associated with improved fertilization and pregnancy outcomes compared to general anesthesia. For instance, Aghaamoo et al. reported that spinal analgesia had potential advantages over general anesthesia for achieving IVF success [23]. Similarly, Azmude et al. found comparable pregnancy rates between patients receiving spinal and general anesthesia, raising the question of whether the general physiological effects of these anesthetics could influence gamete quality [24]. There were studies reporting that the combination of hypnotics and opioids was frequently used in in vitro fertilization (IVF) patients and that the risk of adverse effects on oocyte, embryo quality and pregnancy rates was relatively low [25,26]. Studies comparing spinal with general anesthesia in IVF procedures reveal that anesthesia modality may influence not only procedural success but also cellular integrity of gametes [27]. It is well determined that general anesthesia is associated with adverse outcomes in IVF for oocyte retrieval [28]. A study comparing epidural anesthesia, paracervical block, and general anesthesia with nitrous oxide for oocyte retrieval showed that pregnancy rates in women who underwent general anesthesia were significantly lower than in the other two groups [29]. Conscious sedation and analgesia have been advertised as one of several methods for pain relief in IVF; however, none have been compared with spinal anesthesia, and none have evaluated the effects of intravenous anesthetic drugs on the fetus and its survival. However, the fact that blood levels of local anesthetic were very low after administration into the subarachnoid space [30], may be the most likely explanation for the better results in the spinal anesthesia group compared to general anesthesia.

Wang et al. [14] demonstrated that MSV under local anesthesia leads to significant improvements in key spermiogram parameters. The study recorded improvements in sperm concentration, total count and motility that reached statistical significance (all *p* values < 0.05). These findings offer a proof-of-concept that minimizing systemic exposure to anesthetic agents could favorably influence spermiogram outcomes. In patients with high anxiety or in cases where local anesthesia might not be feasible, spinal anesthesia, as indicated by ART studies, may serve as a suitable alternative that balances intraoperative comfort with optimized reproductive outcomes.

Our study has several important limitations. The retrospective design inherently limits causal inference despite our use of propensity score matching. While PSM achieved good covariate balance (SMD < 0.1), unmeasured confounders may have influenced both anesthesia selection and outcomes. The Rosenbaum bounds analysis (Γ = 1.6) suggests moderate robustness to hidden bias but does not eliminate residual confounding. Another point to consider is that the SA group (*n* = 38) is substantially smaller than recommended by our a priori power calculation (*n* = 64), increasing the risk of Type II error. This sample size also reflects the clinical reality at our institution where GA is strongly preferred by both patients and surgeons. Furthermore, selection bias related to follow-up completion is a concern because only patients with complete 24-month data were included, potentially excluding those who achieved early pregnancy and discontinued follow-up semen analyses or those dissatisfied with outcomes. We did not systematically document reasons for loss to follow-up or compare baseline characteristics between completers and non-completers, which could introduce bias in both directions. Additionally, despite standardized anesthetic protocols, we did not document intraoperative anxiety levels or VAS scores in the SA group. We also did not measure mechanistic biomarkers including oxidative stress markers (reactive oxygen species), hormonal profiles (testosterone, LH, FSH), or sperm DNA fragmentation indices, which would have provided direct evidence for the proposed mechanisms linking anesthesia type to reproductive outcomes. Another important issue is that semen analyses were performed by multiple andrologists, introducing potential interobserver variability despite standardized protocols and quality control measures. Finally, this single-center study was conducted in a specific healthcare setting with particular patient demographics and clinical practices. External validity to other populations, healthcare systems, or anesthetic protocols may be limited.

## 5. Conclusions

In conclusion, this retrospective cohort study with 24-month follow-up found no difference in sperm count (primary outcome) between patients receiving spinal versus general anesthesia for MSV. However, spinal anesthesia was associated with significantly greater improvement in sperm motility from month 3 onwards and higher natural pregnancy rates (42.1% vs. 32.9%). Other sperm parameters were comparable between groups. These associations suggest that anesthesia modality may influence reproductive outcomes following MSV, though causality cannot be established from this retrospective cohort study. Prospective randomized controlled trials with larger sample sizes, mechanistic biomarker measurements (oxidative stress, hormonal profiles, sperm DNA fragmentation), and standardized female partner evaluation are needed to confirm these findings and elucidate the underlying mechanisms.

## Figures and Tables

**Table 1:** Baseline characteristics and preoperative parameters of patients undergoing varicocelectomy.

Characteristics	General Anesthesia (*n* = 380)	Spinal Anesthesia (*n* = 38)	*p* Value	SMD
Age and anthropometric data				
Age (years)	34.15 ± 9.61	33.55 ± 9.66	0.711	0.062
BMI (kg/m^2^)	26.42 ± 4.85	27.79 ± 5.03	0.096	0.089
ASA physical status, *n* (%)				
I	186 (48.9)	17 (44.7)	0.876	0.074
II	156 (41.1)	17 (44.7)		
III	38 (10.0)	4 (10.5)		
Comorbidities, *n* (%)				
Smoking	112 (29.5)	11 (28.9)	0.946	0.013
Hypertension	68 (17.9)	7 (18.4)	0.936	0.013
Diabetes mellitus	42 (11.1)	4 (10.5)	0.919	0.019
Dyslipidemia	54 (14.2)	5 (13.2)	0.866	0.029
Varicocele characteristics, *n* (%)				
Grade II	228 (60.0)	22 (57.9)	0.924	0.058
Grade III	152 (40.0)	16 (42.1)		
Bilateral	342 (90.0)	34 (89.5)	0.918	0.016
Preoperative semen parameters				
Sperm Count (million/mL)	11.54 ± 2.04	11.56 ± 2.06	0.931	0.033
Sperm Motility (%)	32.65 ± 4.31	32.62 ± 4.25	0.932	0.007
Sperm Morphology (%)	2.97 ± 0.56	2.99 ± 0.62	0.810	0.041
Semen Volume (mL)	2.70 ± 0.70	2.84 ± 0.68	0.082	0.068
Duration of infertility				
Months, median (IQR)	24 (18–36)	26 (19–38)	0.842	0.52

Continuous variables are presented as mean ± standard deviation unless otherwise specified. Categorical variables are presented as number (percentage). BMI: Body Mass Index; ASA: American Society of Anesthesiologists; IQR: Interquartile range; SMD: Standardized Mean Difference. All SMD values < 0.1 indicate excellent balance between groups after propensity score matching.

**Table 2:** Comparison of perioperative complications and recovery parameters between the groups.

Complications	General Anesthesia (*n* = 380)	Spinal Anesthesia (*n* = 38)	*p* Value
Intraoperative Events, *n* (%)			
Bradycardia (<50 bpm)	19 (5.0)	3 (7.9)	0.442
Hypotension (MAP < 50 mmHg)	24 (6.3)	1 (2.6)	0.714
Bleeding (>50 mL)	13 (3.4)	0 (0.0)	0.614
No complications	324 (85.3)	34 (89.5)	0.624
Postoperative Events, *n* (%)			
Nausea/Vomiting	62 (16.3)	8 (21.1)	0.605
PDPH	0 (0.0)	3 (7.9)	<0.001
Urinary retention	7 (1.8)	6 (15.8)	<0.001
Scrotal hematoma	5 (1.3)	0 (0.0)	0.721
Recovery Parameters			
Time to ambulation (h)	6.2 ± 1.8	4.1 ± 1.2	<0.001
Length of hospital stay (h)	28.4 ± 6.3	24.7 ± 5.1	0.002
Morphine consumption in the first 24 h of the postoperative period (mg)	8.4 ± 5.7	2.8 ± 3.2	<0.001
VAS pain score at 6 h	3.8 ± 1.4	2.1 ± 0.9	<0.001
VAS pain score at 24 h	2.3 ± 0.8	1.7 ± 0.6	0.004

Values are presented as number (percentage) or mean ± standard deviation. MAP: mean arterial pressure; PDPH: Post-dural puncture headache; VAS: Visual analog scale.

**Table 3:** Postoperative outcomes and improvement rates in sperm parameters.

Parameter	General Anesthesia (*n* = 380)	Spinal Anesthesia (*n* = 38)	Mean Difference (95% CI)	*p* Value
Postoperative values (at 24th month after surgery)				
Sperm Count (million/mL)	17.48 ± 2.63	18.29 ± 2.53	0.81 (−0.09 to 1.71)	0.076
Sperm Motility (%)	46.39 ± 4.73	49.55 ± 4.74	3.16 (1.51 to 4.81)	0.0002
Sperm Morphology (%)	4.45 ± 0.64	4.67 ± 0.71	0.22 (−0.01 to 0.45)	0.058
Semen Volume (mL)	3.18 ± 0.72	3.30 ± 0.66	0.12 (−0.29 to 0.31)	0.096
Improvement from Baseline (%) *				
Sperm Count	56.84 ± 28.41	63.63 ± 30.12	6.79 (−5.71 to 19.29)	0.285
Sperm Motility	44.76 ± 21.83	54.46 ± 23.65	9.70 (1.12 to 18.28)	0.027
Sperm Morphology	55.35 ± 58.42	63.95 ± 61.78	8.60 (−14.52 to 31.72)	0.468
Semen Volume	17.77 ± 32.45	16.19 ± 31.87	−1.58 (−16.52 to 8.94)	0.553
Pregnancy rate, % (*n*/N)	32.9 (125/380)	42.1 (16/38)	9.2	0.031
Achievement of WHO reference values, *n* (%)				
Sperm Count ≥ 15 million/mL	312 (82.1)	34 (89.5)	N/A	0.368
Motility ≥ 40%	342 (90.0)	37 (97.4)	N/A	0.229
Morphology ≥ 4%	289 (76.1)	32 (84.2)	N/A	0.318
Volume ≥ 1.5 mL	371 (97.6)	37 (97.4)	N/A	0.947

Continuous variables are presented as mean ± standard deviation. * Improvement percentages were calculated as = [(postoperative value − preoperative value)/preoperative value] × 100. WHO: World Health Organization; CI: confidence interval; N/A: not applicable.

## Data Availability

The data that support the findings of this study are available from the corresponding author upon reasonable request.

## Abbreviations

The following abbreviations are used in this manuscript:

ART	Assisted reproductive technology
ASA	American Society of Anesthesiologists
BMI	Body mass index
GA	General anesthesia
IVF	In vitro fertilization
MSV	Microscopic subinguinal varicocelectomy
SA	Spinal anesthesia
WHO	World Health Organization
